# ECAPA-TDNN based online discussion activity-level evaluation

**DOI:** 10.1038/s41598-024-63874-3

**Published:** 2024-06-26

**Authors:** Hongbo Kang, Botao He, Ruoyang Song, Wenqing Wang

**Affiliations:** Present Address: School of Automation, Xi’an University of Posts and Telecommunications, Xi’an, China

**Keywords:** ECAPA-TDNN, Voiceprint recognition, Audio segmentation, Activity-level evaluation model, Online education, Engineering, Mathematics and computing

## Abstract

With the continuous development and application of online interactive activities and network transmission technology, online interactive behaviors such as online discussion meetings and online teaching have become indispensable in people’s studies and work. However, the effectiveness of working with online discussions and feedback from participants on their conference performance has been a major concern, and this is the issue examined in this post. Based on the above issues, this paper designs an online discussion activity-level evaluation system based on voiceprint recognition technology. The application system developed in this project is divided into two parts; the first part is to segment the online discussion audio into multiple independent audio segments by audio segmentation technology and train the voiceprint recognition model to predict the speaker’s identity in each separate audio component. In the second part, we propose a linear normalized online meeting activity-level calculation model based on the modified main indexes by traversing and counting each participant’s speaking frequency and total speaking time as the main indexes for activity-level evaluation. To make the evaluation results more objective, reasonable, and distinguishable, the activity score of each participant is calculated, and each participant’s activity-level in the discussion meeting is derived by combining the fuzzy membership function. To test the system’s performance, we designed an experiment with 25 participants in an online discussion meeting, with two assistants manually recording the discussion and a host moderating the meeting. The results of the experiment showed that the system’s evaluation results matched those recorded by the two assistants. The system can fulfill the task of distinguishing the level of activity of participants in online discussions.

## Introduction

As a particular form of online discussion meeting, online teaching solves the problem of needing help to gather in the classroom at specific times and saves time for teachers and students. However, the disadvantages of online education are the need for more interaction between teachers and students and the lack of effective monitoring and teacher feedback on students’ classroom status. A.B. Sulaiman^1^ presented experiential learning online instruction proposals during COVID-19. Online teaching cultivates students’ self-learning habits^2^. Still, the lack of cooperative learning among students, the lack of self-management skills among students, and the inability of teachers to obtain feedback on students’ learning activities in class also raise questions about the effectiveness of online teaching. In offline teaching scenarios, teachers mainly use students’ vocal and eye contact information to capture how active students are in the classroom. However, in the online teaching process, teachers spend all their time in the lecture and thus only have access to some students’ listening statuses. In the mainstream online teaching software used today, teachers can only access students’ presence by (1) Using the online classroom roll call function to find out if students are present in the classroom; (2) Posting questions in class for a limited period to obtain students’ listening status; (3) observe the content and number of pop-ups during discussion questions to obtain the effectiveness of the lesson. The above methods need to be more comprehensive to obtain the classroom performance of all students, so the online teaching platform needs an activity-level evaluation function to assist teachers in observing students’ classroom performance.

For problems related to online discussion sessions, S Debbarma^3^ proposed the application of voiceprint recognition technology in online examinations. Zafar^4^ proposed real-time speaker identification in online video conferencing. Zheng^5^ proposed the design and implementation of a class roll call system based on i-vector voiceprint recognition, which helps teachers record absenteeism, impersonation, and other situations during the teaching process. Chang^6^ proposed a speaker automatic recognition based on the MFCC(Mel-scale Frequency Cepstral Coefficients) Gaussian mixture model applied to the analysis of the teacher-student question-and-answer interactions in research classrooms. Huang^7^ proposed the analysis of classroom interaction behavior based on voice recognition, i.e., the analysis of student’s performance in the school through voice recognition of classroom speech. The above studies show that vocal recognition technology can take on the task of identity recognition and authentication in online teaching and meetings, and it should also be feasible if voiceprint recognition technology is applied to online education activity level evaluation, which can achieve the purpose of assisting teachers to complete classroom observation and improve teaching efficiency.

From the point of view of the application of voiceprint recognition technology, compared with the voiceprint recognition technology in exams proposed by S Debbarma and the voiceprint recognition roll call system proposed by Zheng, the system designed in this paper has a wider range of applications, which can be used not only in the field of education but also can be greatly promoted for use in the work conference. Compared with the voiceprint recognition technology in videoconferencing proposed by Zafar, this paper can calculate and produce results on the speaker’s activity in the meeting based on recognizing the speaker. Compared with Huang’s proposed voiceprint recognition technology based on the analysis of classroom interaction behavior, etc., this paper is applied in unlimited locations, not only online or offline can conclude the speaker’s activity. To sum up, the online discussion meeting activity analysis system designed in this study can not only recognize the identity of speakers through speech recognition technology, but also analyze the meeting activities of specific speakers through statistical data, and finally give quantitative scores by combining with the activity calculation model, so the range will be much wider in practical applications.

Like fingerprints, each person’s vocal characteristics and articulation style are unique, and the aspects of a speaker’s articulation and the structure of the vocal tract cannot be successfully replicated through imitation. Voiceprint features^8^ are parameters extracted from the speaker’s audio that represent the audio personality of each speaker, so they can be used as unique characteristics of each person to distinguish between speakers. Voiceprint recognition technology can be technically divided into speaker confirmation and identification technology^9^. Speaker confirmation technology is to give voiceprint features in advance. When a speaker vocalizes, it is compared with the given voiceprint features to determine whether it is the identity of the specified person, which is suitable for one-to-one identity confirmation situations such as intelligent home wake-up, financial sector identity authentication, and public security forensic identity detection. Speaker recognition technology is given multiple voiceprint features when the speaker occurs to distinguish which of the numerous voiceprint features the speaker is commonly used in identity recognition of many-to-one situations.

Kesta et al. of Bell Labs were the first to propose the concept of the voiceprint, and in 1962 they first proposed that voiceprint recognition could be achieved by pattern matching on such a speech spectrogram. Since then, many researchers have conducted research on address spectrograms^10^ and applied more signal-processing methods to speech data to achieve voice recognition, which has extensively promoted the development of voice recognition technology.

Luck first used the cepstrum technique for speaker recognition in 1969 and got better results. Atal et al.^11^ used LPCC (linear predictive cepstrum coefficient) for speaker recognition and improved the accuracy of the recognition coefficient. However, it has the problems of high computational complexity, sensitivity to noise and distortion, and inability to handle non-stationary signals. Since then, researchers have shifted their research focus to the processing of acoustic feature parameters and new pattern-matching methods. LPC^12^ (Linear predictive coding) spectral coefficients, PLP^13^ (Perceptual linear predictive), and other speaker recognition feature parameters have been proposed successively. These methods are widely used because of their advantages such as high efficiency, scalability, and ease of implementation. However, there are some drawbacks, such as high computational complexity, sensitivity to noise and distortion, and sensitivity to parameter settings.

In 1990, Davis^14^ demonstrated that the Merle cepstral coefficients were more accurate in characterizing speech, with higher accuracy, lower error rates, and good robustness compared to other speech features. In 1995, Reynolds^15^ first applied the GMM (Gaussian hybrid model) to fit voiceprint features in audio and achieved good performance and accuracy in text-independent voiceprint recognition experiments with the Gaussian hybrid model. GMM has become the dominant technique in text-independent speaker recognition due to its simplicity, flexibility, effectiveness, and robustness. However, it still has the disadvantages of being sensitive to initial values and outliers, failing for high-dimensional data, and not being able to accurately cluster data whose distribution is non-spherical.

In 2000, Reynolds et al.^16^ proposed an improved scheme based on GMM: the UBM (Universal Background Mode) to reduce the effect of environmental noise on voiceprint recognition. UBM is obtained from a large number of speakers’ speech parameters, which can reflect the average distribution of many people’s speech parameters in the feature space. UBM has a flexible structure and excellent robustness, which drastically improves the speaker recognition performance. UBM also suffers from the problems of hybridity, channel variability, and model complexity.

In 2008, Patrick Kenny proposed a JFA^17^ (Joint Factor Analysis) model for the analysis of voiceprint recognition, which makes assumptions about the feature space of voiceprints and channels, assuming that the two are independent and uncorrelated states. JFA has drawbacks that may lead to degradation of recognition accuracy, limited adaptation to nonlinear variations, and limited ability to handle high-dimensional data when processing speaker information and channel information.

In 2009, Dehak found that it is possible to model the feature information^18^ of a voiceprint recognition simultaneously with the features of the channel using a super vector subspace. This space that models the variability of the speaker and the track simultaneously is the total factor space. The mapping of each speech segment in this space is called the identity vector (i-vector)^19–21^. However, the i-vector’s sensitivity to initial values can cause the model to fail to converge to an optimal solution and have limited ability to handle high-dimensional data. It may not be sensitive enough to changes in speaker information, and i-vector may not be able to accurately extract the speaker’s voice features if they have changed significantly.

In recent years, with the development of deep learning, the introduction of DNNs has greatly reduced the recognition error rate. The algorithm at this time, which can be called the embedding algorithm, still extracts features, but this time the activation units of the final hidden layer of the neural network are extracted as embedding instead of the i-vector as the feature representation of a piece of speech. At this point, the d-vector^22^, x-vector^23^ , and j-vector^24^ models emerge. x-vector system has resulted in significant architectural improvements and optimized training procedures^25^ over the original approach. The topology of the system was improved by incorporating elements of the popular ResNet^26^ architecture. Adding residual connections between the frame-level layers has been shown to enhance the embeddings^27,28^. Whether obtaining the identity vector i-vector in full factor space or extracting the audio embedding through a neural network, essential features are extracted from the speech and then classified or confirmed. The end-to-end^29^ system combines these two ends into one system, integrating feature extraction, data training, and voiceprint and performing systematic evaluation from input to output, significantly different from previous approaches to voiceprint recognition.

Improving upon the original Time Delay Neural Network^30^ (TDNN) architecture is an active area of research. The proposed ECAPA-TDNN^31^ (Emphasized Channel Attention, Propagation, and Aggregation in time delay neural network-based Speaker Verification) architecture significantly outperforms state-of-the-art TDNN-based systems on the VoxCeleb test sets and the 2019 VoxCeleb Speaker Recognition Challenge. ECAPA-TDNN by introducing the SE (squeeze-excitation) module as well as the channel attention mechanism, the solution achieved first place in the international voiceprint recognition competition (VoxSRC2020).

Combining the above problems and techniques, this project designs an online discussion activity analysis system based on voiceprint recognition technology, which can reasonably derive the activity level of each participant in a discussion meeting, and concludes the research problem by applying voiceprint recognition technology to obtain the activity level of the participants in an online discussion meeting, which is studied in this paper. Unlike the multi-speaker identification problem proposed in cocktail parties^32^, since multiple people usually speak online discussions in an orderly manner, the audio of online meetings can be segmented into various speech segments through audio segmentation techniques, with each segment having speaking information of only one person, i.e., the identification problem of classroom audio can be solved through speaker identification, which can efficiently and quickly provide an activity-level of online meeting participants feedback. The speaker’s identity, the frequency of speech, and the total time spent speaking are obtained by analyzing the voiceprint features and comparing them with the voiceprint database. In this application system design process, we need to complete the work of training voice recognition, segmenting conference audio, designing the active-level evaluation formula, designing experiments, and analyzing the system’s feasibility through the experimental results.

The research methodology and line of research in this study is to build on previous work done in the literature on voiceprint recognition technology to validate the direction of the application of voiceprint recognition as well as the feasibility of the application of voiceprint recognition technology in online discussion meetings. Applying voiceprint recognition technology to online discussion meetings, combined with the activity calculation model proposed in this study, the active status of online discussion meeting attendees is obtained, and the activity is presented as a quantitative score. First, we carefully read the papers related to the development history and possible future directions of voiceprint recognition technology, summarized the characteristics and application scenarios of each method in voiceprint recognition technology, and finally chose the ECAPA-TDNN as the method used in this study. Based on the realization of voiceprint recognition, we designed audio segmentation, activity calculation models, and online discussion meeting experiments based on the characteristics of online discussion meeting audio.

This project uses the Action-Research^33^ methodology. The following steps were taken to apply the methodology: “Definitional problem—Understanding the context—User research—Design thinking—Implementation and testing—Summary and reflection—Knowledge translation”:

- *Definitional problem* The research question was clarified to obtain participant activity ratings from online discussion sessions.
- *Understanding the context* An understanding of the relevant context of the problem and existing research findings, and the integration of voiceprint recognition technology with online discussion sessions as an innovation in the research.
- *User research* Identifying the main users of the problem under study as students, teachers, and staff using online conferencing software.
- *Design thinking* A systematic design of the research problem was carried out, identifying technical tools such as voiceprint recognition techniques, audio segmentation techniques, and activity analysis models needed to solve the problem.
- *Implementation and testing* The training of the voiceprint recognition model, the use of audio segmentation technology, and the design of the activity analysis model were implemented, and online discussion meeting experiments were carried out according to the system verification requirements, which verified the feasibility and effectiveness of the design of this topic.
- *Summary and reflection* The results and experience of the experiment are summarized, and the design is reflected upon, with suggestions for improvement.
- *Knowledge translation* Applying the designed system to online discussion meetings to improve the discipline and efficiency of online meetings.

Obviously, the results of the online discussion meeting experiment confirm the feasibility of applying voiceprint recognition technology in the direction of meeting activity analysis and provide a viable solution to our research problem.

## Literature review

**Figure 1:**
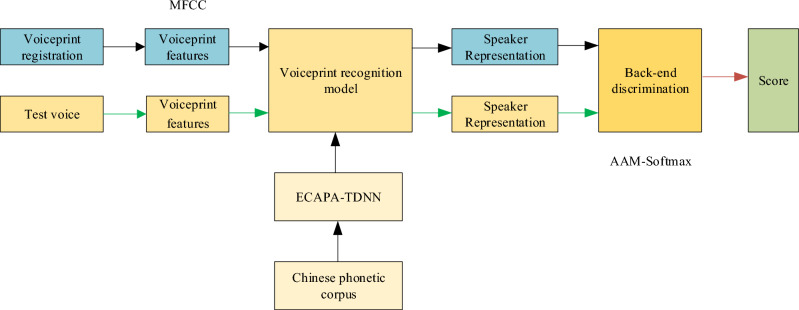
The implementation of voiceprint recognition consists of voiceprint feature pre-processing, voiceprint recognition models, and back-end discriminations respectively.

Voiceprint recognition technology identifies speakers by analyzing the information on speaker characteristics contained in the speech signal and consists of two main stages^34,35^: training and recognition: In the training phase, Combined with the ECAPA-TDNN voiceprint recognition model and a Chinese speech corpus is used to train the voiceprint recognition model; in the recognition phase, the voiceprint features of the audio to be recognized and the registered audio are extracted respectively, the speaker representation is output using the voiceprint recognition model, and the recognition result is derived by back-end discrimination. The basic principle is shown in Fig. 1, where voiceprint feature extraction, voiceprint recognition model, and back-end discriminations are the critical technologies in speaker recognition.

### Extracting MFCC features

MFCC^36^ is a commonly used voiceprint feature; Mel frequency is proposed based on the acoustical properties of the human ear, which is in non-linear correspondence with Hz frequency. MFCC is an inverse spectral parameter extracted in the frequency domain of the Mel scale and calculated to obtain Hz spectral features. MFCC is mainly used for feature extraction and dimensionality reduction of speech data, describing the non-linear characteristics of human ear frequencies, which is more in line with the acoustic characteristics of the human ear and enables better representation of audio signals. Its relationship with $f$(frequency) can be approximated by the following equation:

1 $$\begin{array}{c}Mel\left(f\right)=2595\times \mathit{lg}\left(1+\frac{f}{700}\right)\end{array}$$

**Figure 2:**
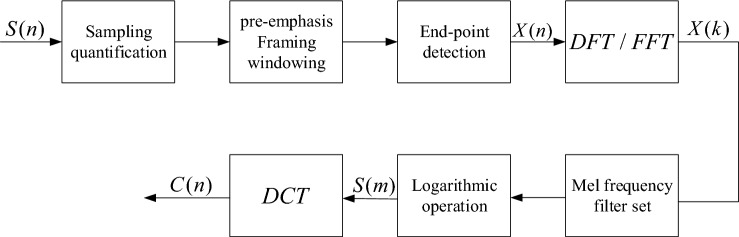
The speech signal S(n) is sampled and quantized, digitised on the time and amplitude axes at a specific sampling rate and accuracy, and turned into a time-domain signal X(n) by a pre-processing method. X(n) is complemented by several zeros to form a sequence of length N. The time domain signal is then converted into a linear spectrum X(k) using the DFT (Discrete Fourier Transform) or FFT (Fast Fourier Transform). X(k) is converted into a Mel spectrum by a Mel frequency filter bank and the logarithmic energy is taken for Mel to obtain the logarithmic spectrum S(m). S(m) is subjected to the DCT (Discrete Cosine Transform) to obtain the MFCC parameter C(n).

The MFCC extraction process is shown in Fig. 2:

The MFCC parameter extraction process is as follows:

- The initial speech signal $S(n)$ is sampled and quantized, digital in the time and amplitude axes at a particular sampling rate and accuracy, and turned into a time domain signal $X(n)$ by pre-emphasis, framing, windowing, and other pre-processing methods.
- The time domain signal $X(n)$ is complemented by several zeros to form a sequence of length N. $DFT$(Discrete Fourier Transform) or $FFT$(Fast Fourier Transform) is then used to convert the time domain signal to a linear frequency spectrum $X(k)$.
- The linear spectrum $X(k)$ is converted to a Mel spectrum by a Mel frequency filter bank, and the logarithmic energy is taken for Mel to obtain the logarithmic spectrum $S(m)$.
- The logarithmic spectrum $S(m)$ is $DCT$(Discrete cosine transformed) to obtain the MFCC parameter $C(n)$.

### Constructing ECAPA-TDNN

**Figure 3:**
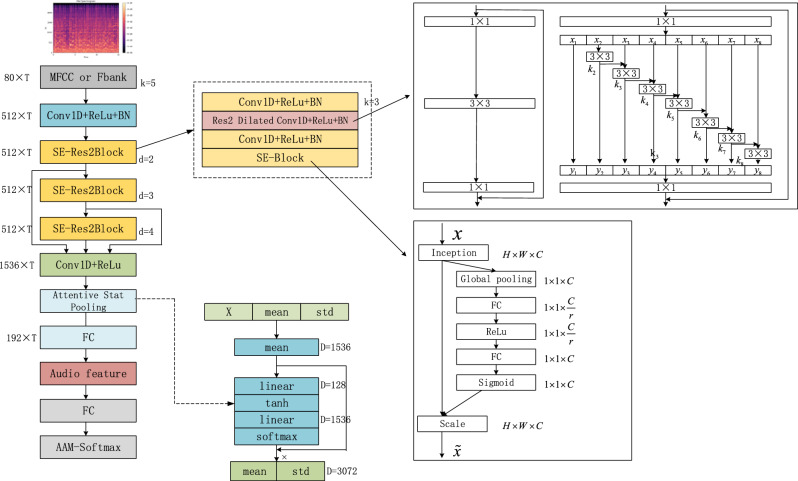
The model inputs 80-dimensional F-bank or MFCC features of length T in this network structure. Where k denotes the kernel size (k = 3 convolution is an inflated convolution, which is a one-dimensional convolution), d represents the expansion spacing of the Conv1D layers or SE-Res2Blocks, C denotes the channel of the intermediate feature map, T denotes the time dimension, and S denotes the number of speakers.

In this system, we use the mainstream voiceprint recognition model ECAPA-TDNN, the model structure of ECAPA-TDNN is shown in Fig. 3 below,

The model inputs 80-dimensional F-bank or MFCC features of length T in this network structure. Where k denotes the kernel size (k = 3 convolution is an inflated convolution, which is a one-dimensional convolution), d represents the expansion spacing of the Conv1D layers or SE-Res2Blocks^37^, C denotes the channel of the intermediate feature map, T denotes the time dimension, and S denotes the number of speakers.

- The first part is a time-delayed neural network module consisting of a one-dimensional convolution, an activation function, and batch normalization^38^;
- The second part will have N layers of SE-Res2Net (squeezed residual excitation module); the number of layers is the number of channels minus two, there are five layers in the specific implementation, so there are three layers of SE-Res2Net modules here, as well as a null convolution.
- The third part of the layer, the TDNN module, serves as a multi-layer feature fusion, where the input is stitched together from the output of each previous layer of the SE-Res2Net module.
- The fourth part is the Attentive Statistical Pooling layer^39^, which is used for the probabilistic pooling of the output.
- The five parts are fully connected layers plus a batch normalization layer used to perform a linear transformation of the final features.
- The final layer is AAM-Softmax^25^ (Additive Angular Margin Loss for Deep Face Recognition), which first normalizes the feature vectors and weights and adds an angular interval $m$ in the $cos\theta$ range to maximize the classification bounds in the angular space $\theta$ to make the classification tighter and is used to classify the output by the number of speakers.
  2 $$\begin{array}{c}{\mathcal{L}}_{AAM}=-\frac{1}{m}\sum_{i=1}^{m}\text{log}\frac{{e}^{s\cdot (\text{cos}({\theta }_{{y}_{i}}+m))}}{{e}^{s\cdot (\text{cos}\left({\theta }_{{y}_{i}}+m\right))}+{\sum }_{j=1,j\ne {y}_{i}}^{n}{e}^{s\cdot \text{cos}{\theta }_{j}}}\end{array}$$

where $i$ is the number of classifications, ${\theta }_{{y}_{i}}$ is the angle between the input features and the weights, $m$ is the corner margin penalty on the target angle ${\theta }_{{y}_{i}}$, and $s$ is the feature vector normalization constant.

ECAPA-TDNN is based on the traditional TNDD model with three optimized improvements:

- 1-Dimensional Squeeze–Excitation Res2Blocsk

The first component of Squeeze–Excitation is the squeeze operation, which generates descriptors for each channel and consists only of computing the average vector of frame-level features across the time domain: the frame-level features for each frame are averaged in time, and the input features are $[N,C,L]$, which are compressed to $[N,C,1]$ by averaging pooling.

3 $$\begin{array}{c}Z=\frac{1}{T}\sum_{t}^{T}{h}_{t}\end{array}$$

where $N$ is the batch size, $L$ is the number of feature frames^40^, $C$ is the channel, $Z$ is the average vector of frame-level features across the time domain, $T$ denotes the time dimension, and ${h}_{t}$ is the activation value of the last frame layer at time $t$.

The weight of each channel is calculated by using the operator in $Z$ in for the excitation operation:

4 $$\begin{array}{c}s=\sigma \left({W}_{2}f\left({W}_{1}z+{b}_{1}\right)+{b}_{2}\right)\end{array}$$

where $\sigma (\cdot )$ denotes the sigmoid function, $f\left(\cdot \right)$ denotes the nonlinear function, ${W}_{1}\in {\mathbb{R}}^{R\times C},{W}_{2}\in {\mathbb{R}}^{C\times R}$, $R$ is the reduced dimension, and $C$ is the number of input channels.

The vectors s obtained in the Excitation operation are weights between 0 and 1. These weights are multiplied by the original input after each channel calculation to get the estimated output:

5 $$\begin{array}{c}\widetilde{{h}_{c}}={s}_{c}{h}_{c}\end{array}$$

where ${s}_{c}$ is the weight of each channel, ${h}_{c}$ is the original input, and $\widetilde{{h}_{c}}$ is the estimated output.

**Figure 4:**
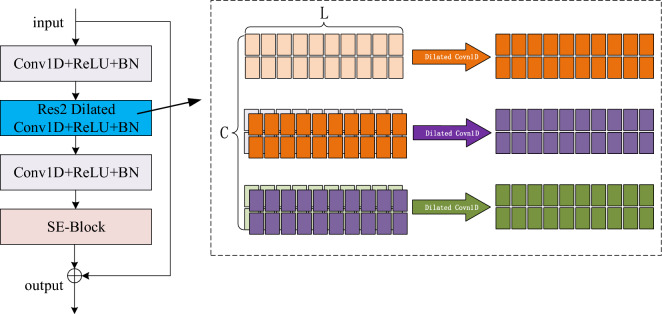
The first part of the squeeze-excitation is the squeeze operation, which generates descriptors for each channel and consists only of computing the average vector of frame-level features over the entire time domain: the frame-level features are averaged in time for each frame, and the input features are [N,C,L], compressed by averaging pooling to [N,C,1]. where {N} is the batch size, {L} is the number of feature frames, and {C} is the number of channels.

The Squeeze-Excitation Res2Block architecture is shown in Figure 4:

(b) Multi-layer feature aggregation and summation

Using a multilayer complementary approach, using all the outputs of SE-Res2Blocks and the initial convolutional layer as input to each frame layer block^41^, this is achieved by defining the residual join of each SE-Res2Block as the sum of the outputs of all previous blocks, choosing the sum of the feature mappings rather than the tandem join to limit the model parameter counts.

(c) Channel-and context-dependent statistics pooling

Different weights are given to each frame through the attention mechanism.

6 $$\begin{array}{c}{e}_{t,c}={v}_{c}^{T}f\left(W{h}_{t}+b\right)+{k}_{c}\end{array}$$

where ${h}_{t}$ represents the frame at moment $t$, $W\in {\mathbb{R}}^{R\times C}, b\in {\mathbb{R}}^{R\times 1}$, $R$ is the reduced dimension, $C$ is the number of input channels, ${e}_{t,c}$ is the scalar fraction, $f\left(\cdot \right)$ denotes the nonlinear function, ${v}_{c}$ is the weight of each frame, and ${k}_{c}$ is the bias weight.

7 $$\begin{array}{c}{\alpha }_{t,c}=\frac{\text{exp}\left({e}_{t,c}\right)}{\sum_{t}^{T}\text{exp}\left({e}_{t,c}\right)}\end{array}$$

where ${\alpha }_{t,c}$ is the self-attentive score and ${e}_{t,c}$ is the scalar score.

The mean values were calculated as:

8 $$\begin{array}{c}\widetilde{{\mu }_{c}}=\sum_{t}^{T}{\alpha }_{t,c}{h}_{t,c}\end{array}$$

where ${\alpha }_{t,c}$ is the self-attention score, $\widetilde{{\mu }_{c}}$ is the weighted average vector, and ${h}_{t,c}$ is the activation value at moment $t$.

The standard deviation is calculated as:

9 $$\begin{array}{c}\widetilde{{\sigma }_{c}}=\sqrt{\sum_{t}^{T}{\alpha }_{t,c}{h}_{t,c}^{2}-}{\widetilde{\mu }}_{c}^{2}\end{array}$$

where $\widetilde{{\sigma }_{c}}$ is the weighted standard deviation, ${\alpha }_{t,c}$ is the self-attentive score, $\widetilde{{\mu }_{c}}$ is the weighted mean vector and ${h}_{t,c}$ is the activation value at moment $t$. The final output of the pooling layer is obtained by connecting the vectors of weighted mean $\mu$ and weighted standard deviation $\sigma$.

### Voiceprint recognition model training

The dataset used for this model training is a Chinese speech corpus, and the dataset is available at https://gitee.com/kuangdd/zhvoice. This dataset consists of 8 open source datasets containing information on three aspects: text, speech, and speaker, and can be applied to various speech-related tasks. It is processed by noise reduction and de-muting, which reduces noise interference and unnaturalness caused by incoherent speaker speech compared to the original data. It is more precise and more in line with the natural environment of online discussion meetings. The audio dataset has approximately 3200 speakers, 900 h of audio, and 1.13 million text entries, with a total of roughly 13 million words, and can be used for various speech-related tasks. The specific information on the Chinese speech corpus is as follows:

1. The Zhaidatatang corpus is an open Chinese Mandarin telephone speech corpus with a corpus length of about 145 h, recorded by 600 speakers from all regions of China with an even distribution of gender and age using Android (16 kHz, 16-bit) and iOS (16 kHz, 16-bit) cell phones in a quiet recording environment containing background noise that does not affect speech recognition, and the speech material is phonetically balanced spoken utterances. The recording environment was quiet and contained background noise that did not affect speech recognition, and the speech material was phonetically balanced spoken utterances.
2. The Zhaishell corpus was recorded by 400 people from different regions of China with different accents using high-fidelity microphones, Android-powered phones, and iOS-powered phones with a uniform sample rate down to 16 kHz.
3. The Zhbznsyp corpus is a library of Chinese standard female voices, recorded by a woman using standard Mandarin in a professional studio for a total of 12 h of audio data.
4. The Zhmagicdata corpus was recorded by 1000 speakers with different accents from different regions of China, using cell phone recordings, and recording scenarios including interactive Q&A, spoken text messaging, music search, etc. The Zhmagicdata corpus is a collection of speakers with different accents from different regions of China.
5. The Zhprimewords corpus was recorded by 296 native Chinese speakers via smartphones, with a transcription accuracy higher than 98% and a confidence level of 95%, and the mapping between paraphrases and utterances is provided in JSON format.
6. The Zhspeechocean corpus is a 10-h long corpus recorded simultaneously by 10 men and 10 women using 4 different microphones.
7. The Zhstcmds corpus contains more than 100,000 voice files containing mainly online voice chat and intelligent voice control utterances.
8. The Zhthchs30 corpus is a classic Chinese speech dataset containing more than 10,000 speech files, recorded over a period of about 40 h with a single carbon microphone, with a focus on article verses and all female voices.

**Figure 5:**
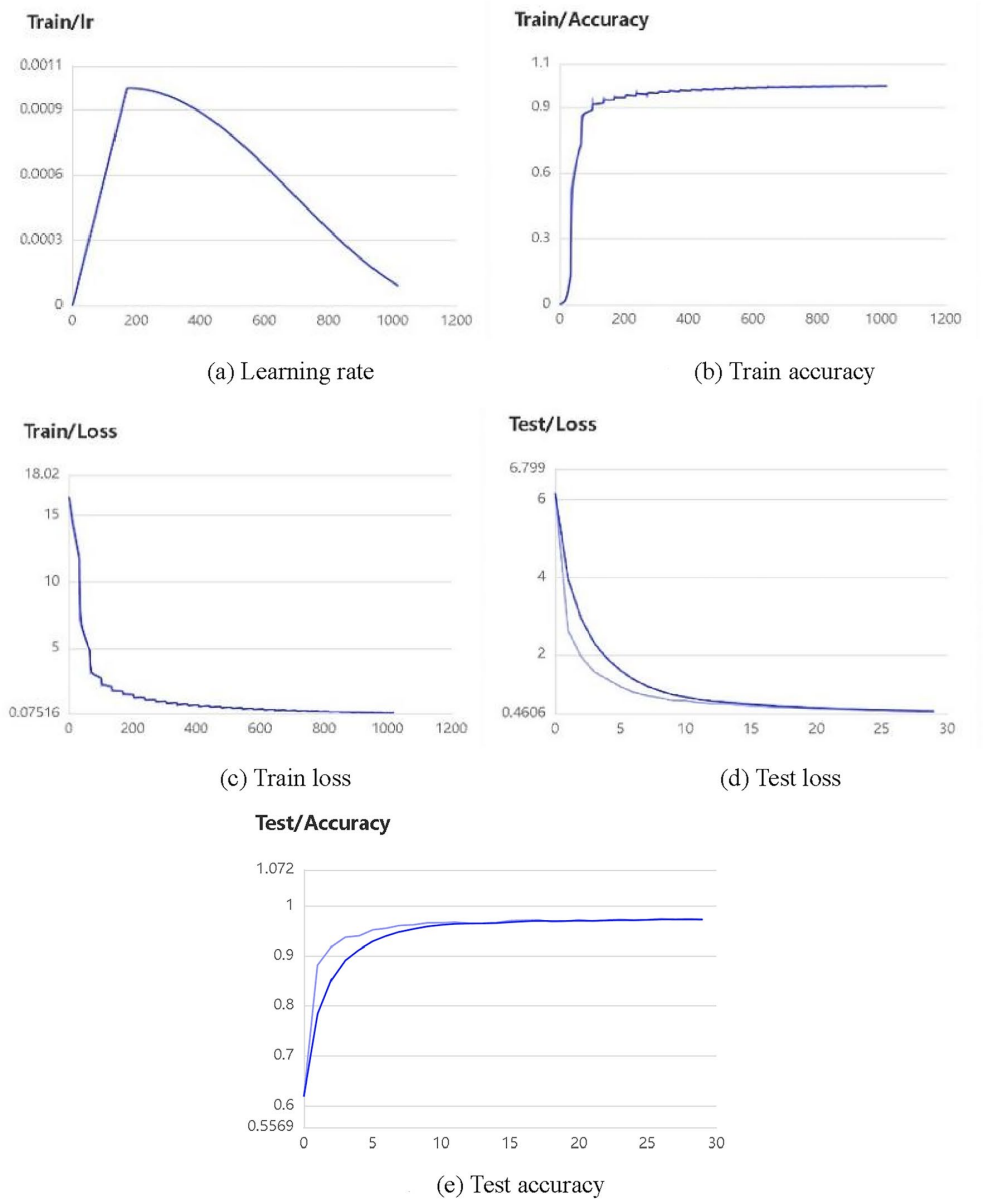
(**a**-**c**) show the change curves of learning efficiency, accuracy, and loss during the training process, respectively, and **d**, **e** show the shift curves of loss and accuracy during the testing process respectively.

We use ECAPA-TDNN as a training model for voiceprint and use MFCC to acquire voiceprint features. The data enhancements used are random cropping, adding background noise, adjusting speech rate, adjusting volume, and SpecAugment^42^. In this case, adding background noise requires storing multiple noise files in a specified folder. The model training parameters are configured as shown in Table 1. During the training process, the training log is saved using Visual-DL, and the training results can be viewed by starting Visual-DL; the training results are shown in Fig. 5.

**Table 1:** Model training parameters configuration.

Data enhancement methods	Adding background noise , adjusting volume, adjusting the speed of speech
Training batch sizes	64
Feature extraction methods	MFCC
Gpus use serial numbers	0
Initial learning rates	0.001
Training rounds	30
Number of classifications	3242
Number of threads to read data	8
Minimum audio duration for training	0.5 s
Model saving path	models/
Test data list paths	dataset/test_list.txt
Training data list paths	dataset/train_list.txt
Training models	ECAPA_TDNN

Among them, Fig. 5a–c show the change curves of learning efficiency, accuracy, and loss during the training process, respectively, and Fig. 5d and e show the shift curves of loss and accuracy during the testing process respectively.

After training, the prediction model is saved and used to predict the voiceprint features in the test set. The voiceprint features are used for a one-by-one comparison with thresholds from 0 to 1 and controlled in steps of 0.01 to find the best threshold and calculate the accuracy to complete the model evaluation, and the model evaluation results are: the classification accuracy is 0.9608, and the maximum accuracy of the two-by-two comparison when the threshold is 0.58 is 0.9998.

**Figure 6:**
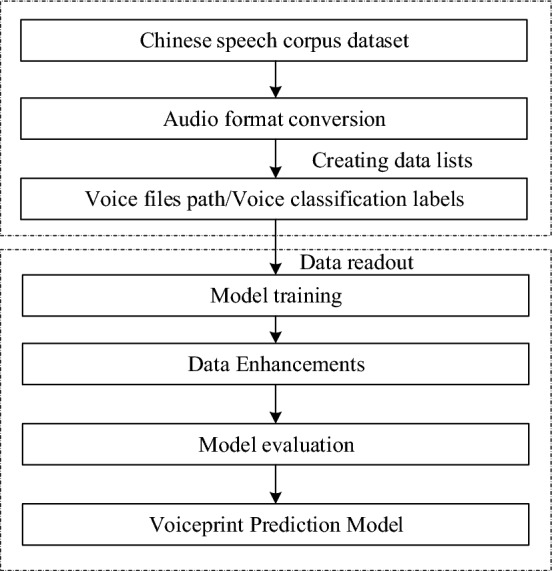
Process of training a vocal recognition model from a Chinese speech corpus dataset.

The voiceprint recognition model training is divided into two parts: data set processing and model training, and the voiceprint recognition model training flow chart is shown in Fig. 6.

## Activity-level evaluation system

### System solutions

**Figure 7:**
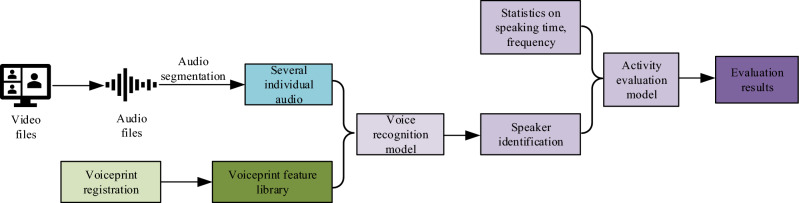
Analysis of student activity in online teaching through voice recognition.

The system first converts the video file of the online discussion session into an audio file, uses the Logmmse noise reduction algorithm for audio noise reduction, and finally splits the audio file into multiple audio segments containing individual speaker information to achieve voiceprint recognition before putting the registered voiceprint features into a unified folder for management. After completing the above tasks, the segmented multiple audios are compared with the voiceprint features in the voiceprint feature library one by one using a vocal recognition model to achieve authentication. The system flow chart for classroom activity-level analysis is shown in Fig. 7.

The activity-level evaluation system designed allows for the following functions:

- *Voiceprint registration* record 3–5 s of audio of a single person speaking, capture the voice features, and store them in the voice feature library.
- *Voiceprint recognition* input two voice files, obtain feature data by prediction function, and use feature data to find diagonal cosine similarity, based on which one-to-many speaker recognition is achieved.
- *Audio splitting* split long audio into multiple independent audio segments by setting the silence decibel and silence time in the audio.
- *Activity-level evaluation* traversal to get the speaker activity-level evaluation indicators and obtain the activity-level evaluation results utilizing the activity-level calculation model and the membership function.

### Voiceprint recognition

Voiceprint feature information is registered by recording 3–5 s of text-independent audio, converting the recorded speaker audio into vocal feature data, and storing it in the verbal feature database under a separate name. Registration is a prerequisite for voiceprint comparison and voiceprint recognition. In the process of voiceprint recognition, if the voiceprint feature database does not contain the voiceprint feature information of the speaker to be recognized, or if it does not reach the similarity threshold in the voiceprint feature database, the identity of the speaker will not be recognized. In addition, this system also implements a query function for the voiceprint feature library to check whether the voiceprint features of the speaker to be identified exist in the library. The principle of the voiceprint comparison is to calculate the diagonal cosine of the two speakers’ representations utilizing AAM-Softmax. The result is the similarity between the audio of the speaker to be recognized and the audio in the voiceprint feature library.

The model evaluation results show that the highest accuracy is achieved at a similarity threshold of 0.58. When the diagonal cosine value calculation exceeds the similarity discrimination threshold, the two audio segments can be confirmed as the same person’s voice. As can be seen from the example of the experimental setup, when the similarity judgment threshold is set to 0.58, the vocal pattern comparison results of student1 and student2 are determined to be not the same person, and the similarity is only 0.1807.

Based on the realization of the voiceprint comparison, speaker recognition can be realized for the audio recorded in real time. The MFCC vocal features of the speaker to be recognized are first extracted, and the vocal features from the loaded vocal feature library are used to obtain the speaker representation data employing a voiceprint recognition model. Finally, the diagonal cosine values of the speaker to be recognized and the voiceprint feature library are calculated one by one, and if the maximum calculation result is greater than the similarity discrimination threshold, the identity of the speaker can be judged to be the user corresponding to the highest diagonal cosine value. From the example set out in the experiment, it can be seen that at a similarity judgment threshold of 0.58, the person to be recognized records 5 s of audio, and the recognition results are shown in Table 2:

**Table 2:** Identification results.

Identification results	Similarity
Host	0.8654
Participants1	0.8832
Participants2	0.7549
Participants3	0.8264
Participants4	0.7359
Participants5	0.7063
Participants6	0.7455
Participants7	0.8554
Participants8	0.6814
Participants9	0.7836
Participants10	0.8945
Participants11	0.7954

### Audio segmentation

**Figure 8:**
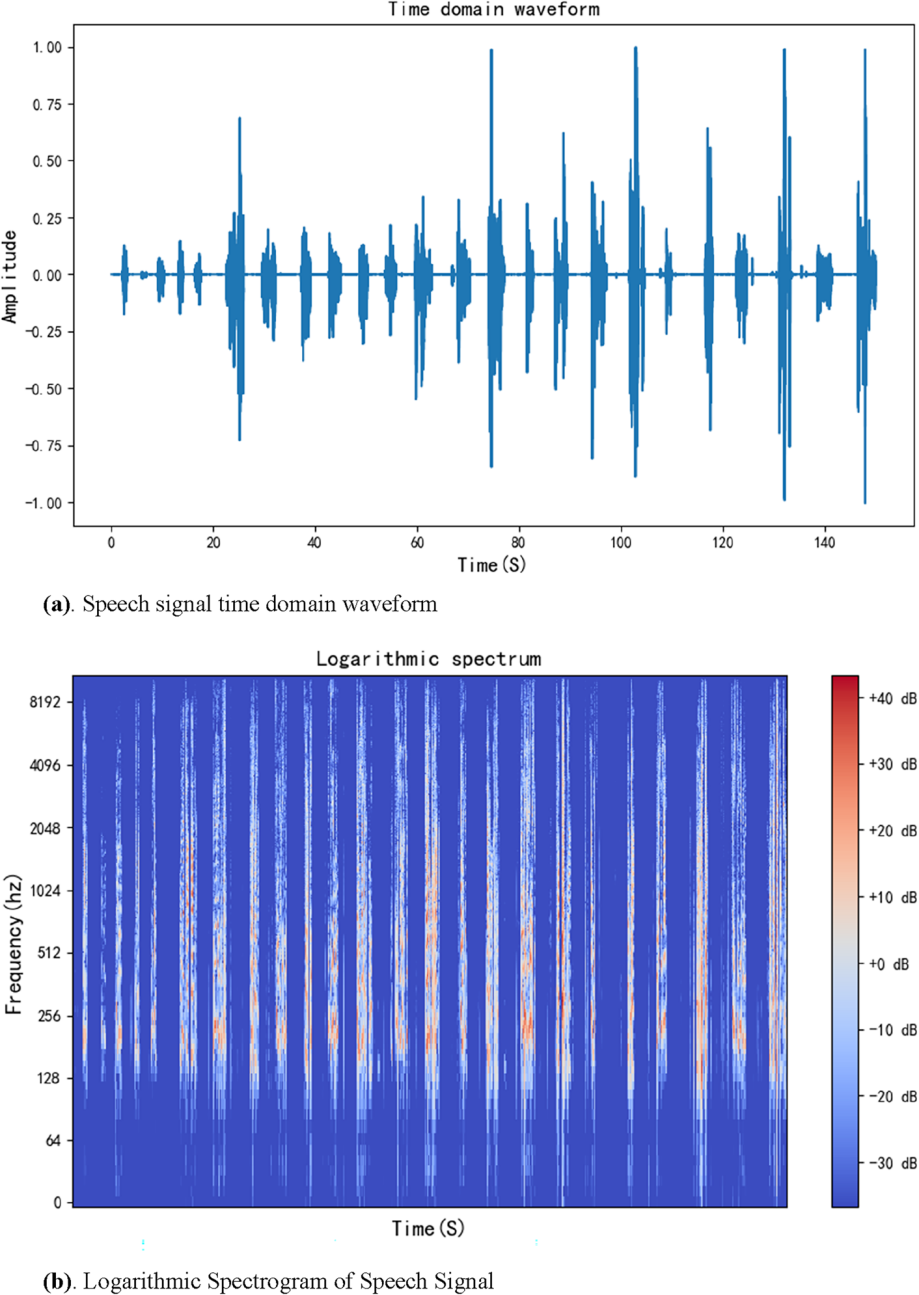
(**a**) The time domain waveform of an audio file with time spaced between each sentence. (**b**) The logarithmic spectrum of a voice file can display time, frequency, and decibel information.

The purpose of audio segmentation^43^ is to segment a piece of online discussion meeting audio into multiple short audio segments with only independent speakers. Based on the discontinuity of online discussion meeting audio, audio less than − 70dbFS is considered silent audio. At the same time, silence longer than 700 ms is split and judged as two speeches, and the segmented multiple audio segments are placed into a voiceprint folder awaiting recognition. During the 150 s of experimental audio segmentation, the audio was sampled at 48,000, the number of channels was 2, the sample width was 2, and the number of samples was 7,200,000. The time of each sample is calculated by the number of samples and the sampling frequency. After normalization and application of the Fourier transform, the audio file’s speech signal time domain waveform and logarithmic Spectrogram of Speech Signal can be drawn, as shown in Fig. 8a and b.

As can be seen from the time domain diagram in the figure, as the timeline moves, the 150 s of audio exhibits multiple segments of different amplitudes, and because of the particular environment of the online discussion session, each segment of amplitude can be seen as a single sentence spoken by the speaker. By analyzing the audio information and setting the silence time and silence decibel, the 150 s of audio is split into 35 short segments of audio, of which 27 are valid, each valid segment of audio is a complete sentence of a separate person, and an invalid blank segments are discarded.

### Activity-level analysis

#### Basic parameters

After the entire audio of the online discussion session was split into multiple short audio segments, the short audio segments were identified with the voiceprint features in the voiceprint library one by one. The identity and speaking duration of each speaker were obtained by traversal, and some of the identification results are shown in Table 3.

**Table 3:** Recognition results.

Student1	Similarity	0.6548	Speaking time	1.298 s
Student2	Similarity	0.5963	Speaking time	2.955 s
Student1	Similarity	0.7023	Speaking time	2.131 s
Student2	Similarity	0.5806	Speaking time	2.627 s

The statistical function was used to obtain two basic parameters of activity-level, namely the number of times each speaker spoke and the total time spent speaking, to complete the preliminary parameters of the online meeting activity-level, as shown in Table 4.

**Table 4:** Statistics of activity-level base parameters.

Speaker	Talking frequency	Total speaking time
Student1	9	21.829 s
Student2	12	29.807 s

#### Activity-level assessment model

A discussion session usually consists of the moderator proposing a topic or question and the participants speaking in turn after they have been authorized to speak. In evaluating participant activity-level, the length, and frequency of presentations are the leading indicators of activity-level, but they cannot be used as absolute criteria. In particular, in teaching discussion scenarios, it is essential to avoid situations where one student speaks too often, to allow a more significant number of students to participate in the classroom discussion, and to orient the classroom discussion in the direction designed by the facilitator. Therefore, the two primary parameters for evaluating the activity-level of participants’ speeches, i.e., the total length of speaking time and frequency, need to be revised. In this paper, we propose a linear normalization-based online discussion activity-level evaluation model. This makes the activity-level evaluation scores closer to the actual situation of online discussions and makes each evaluation result independent and objective.

This project defines the basic parameters of time and frequency:

- Time score

10 $$\begin{array}{c}\left\{\begin{array}{c}{T}_{max}=\mathit{max}\left\{{T}_{1},{T}_{2}\dots {T}_{n}\right\}\\ {T}_{min}=\text{min}\left\{{T}_{1},{T}_{2}\dots {T}_{n}\right\}\\ {P}_{{T}_{score}}=\frac{{T}_{P}-{T}_{min}}{{T}_{max}-{T}_{min}}\end{array}\right.\end{array}$$

where ${T}_{i}(i=\text{1,2}\dots n)$ is the total speaking time of the participants, ${T}_{max}$ and ${T}_{min}$ are the total length of the longest and shortest speeches of all participants, ${T}_{P}$ is the total speaking time of the participants to be evaluated, and ${P}_{{T}_{score}}$ is the time score of the participants to be evaluated.

(b) Frequency score

11 $$\begin{array}{c}\left\{\begin{array}{c}{N}_{max}=\text{max}\left\{{N}_{1},{N}_{2}\dots {N}_{n}\right\}\\ {N}_{min}=\text{min}\left\{{N}_{1},{N}_{2}\dots {N}_{n}\right\}\\ {P}_{{N}_{score}}=\frac{{N}_{P}-{N}_{min}}{{N}_{max}-{N}_{min}}\end{array}\right.\end{array}$$

where ${N}_{i}(i=\text{1,2}\dots n)$ is the frequency of participants’ speeches, ${N}_{max}$, ${N}_{min}$ are the maximum and minimum frequency of speeches among all participants, ${N}_{P}$ is the total length of speeches of participants to be evaluated, and ${P}_{{N}_{score}}$ is the frequency score of participants to be evaluated.

(c) Validity coefficient

12 $$\begin{array}{c}\left\{\begin{array}{c}{T}_{{P}_{average}}=\frac{{T}_{{P}_{X}}}{{N}_{{P}_{X}}}\\ {T}_{average}=\frac{{T}_{sum}}{{N}_{sum}}\\ {P}_{{C}_{\text{effective}}}=\frac{{T}_{{P}_{average}}}{{T}_{average}}\end{array}\right.\end{array}$$

where ${T}_{{P}_{X}}$ is the total speaking time of the participants to be evaluated, ${N}_{{P}_{X}}$ is the speaking frequency, ${T}_{{P}_{average}}$ is the average length of each segment of speech of the participants to be evaluated, ${T}_{sum}$ is the total length of speech of all participants, ${N}_{sum}$ is the total frequency of speech of all participants, ${T}_{average}$ is the average length of each segment of speech of all participants, and ${P}_{{C}_{\text{effective}}}$ is the effective coefficient of participants’ speeches to be evaluated.

(d) Activity score

13 $$\begin{array}{*{20}c} {P_{score} = \left( {\frac{{P_{{T_{score} }} + P_{{N_{score} }} }}{2}} \right) \times P_{{C_{{{\text{effective}}}} }} } \\ \end{array}$$

where ${P}_{score}$ is the participant activity score to be evaluated.

**Figure 9:**
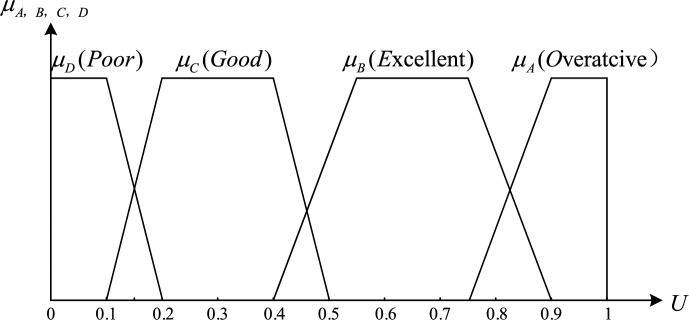
The Fuzzy set membership function classifies student activity in online teaching into four levels: Poor; Good; Excellend; Overactice.

Obviously, if the score is used directly to evaluate the participant’s activity, it is arbitrary and unreasonable. According to an investigation by some teachers, the participant activity should be distinguishable and analytic. In the activity-level assessment model, we introduced a fuzzy set to evaluate the activity-level performance of the participants. Let the activity score ${P}_{score}$ be the domain of the argument, U = $[\text{0,1}]$, and the fuzzy sets $[A,B,C,D]$∈$F(U)$, where $A\hspace{0.17em}$= “overactive”, $B\hspace{0.17em}\hspace{0.17em}$= “excellent activity”, $C\hspace{0.17em}$= “good activity”, $D\hspace{0.17em}$= “poor activity”, and the membership function of each grade of the fuzzy set is shown in Fig. 9:

## Experimental tests

### Design of experiments

1. Because male and female voices sound different, to avoid controversy over the results of the experiment due to the gender ratio of the participants, the gender of the participants was set to be half of each gender, which was set to balance the ratio of male and female participants to ensure that the results of the experiment are reliable and credible.
2. Participants must have clear recording equipment, which will allow for more accurate experimental results.
3. Participants can take part in the online meeting in a quiet, and separate space, thus ensuring that the experimental process is a true reflection of the sound environment of the online meeting.

In the online discussion meeting experiment, participants can use Windows, IOS, and Andriod systems to participate in online discussion meetings in a software called “Tencent Meeting”. Descriptions of the recording devices used by participants: The host used an ASUS-branded ROG series laptop for recording; Participant1, Participant3, Participant9, Participant16, and Participant23 used an iPhone 11 mobile device; Participant8, Participant21, and Participant24 used an iPhone 12 mobile device; Participant5, Participant10, and Participant17 used an iPad pro device; participant2, participant7, and participant12 used a Huawei MATE30pro mobile phone device; participant11, participant18 and participant22 used a Redmi k40 mobile phone device; participant4, participant13, participant15 and participant20 used a Redmi k50 mobile phone device; participant6 and participant19 used the VivoX60 mobile phone device; all of the above participants used the microphone that came with the device to record, while participant14 and participant25 used the airpods wireless Bluetooth headset to continue recording.

To more realistically reproduce the scene of an online discussion meeting, when one participant speaks, the microphone of the rest of the participants will be turned off. The online discussion meeting has no requirement on the location of the participants, they can be at home, in the dormitory of the school, or the office, but the location they are in has the same characteristic of a relatively quiet sound environment. To minimize the impact of spatial noise on the experimental results, participants checked their respective microphones for noise before the start of the online meeting by recording a segment and playing it back to detect the noise, and if the detected noise would affect the system’s ability to carry out voiceprint recognition, the participant would attend the meeting using another device that passed the test. Meanwhile, during an online meeting, when one participant is speaking, the microphones of the others are switched off.

The audio quality of the participants varies because different participants use different recording devices to participate in the online discussion sessions. Of course, in real-life online meetings, there are also situations where audio quality varies from one recording device to another. This is a difficult problem to solve because the recording device determines the audio quality. In this paper, we can only try to keep the audio quality of the final online meeting as consistent as possible with that of the original audio by changing the sampling frequency and the number of samples of the audio. At the same time, attendees will be registering their voices at a single, high-quality recording facility, which ensures that their voice audio will be of relatively high quality.

To confirm that the system designed can effectively analyze the activity-level of online discussion sessions, this experiment was conducted to evaluate the activity-level of an online discussion session of 122 min in length, which had 28 participants, including one moderator (numbered Host), 25 speakers (numbered Participants1, Participants2 …Participants25), and two assistants who are in charge of recording discussion session by hands. Each participant had a discussion of varying frequency and length during the meeting.

**Figure 10:**
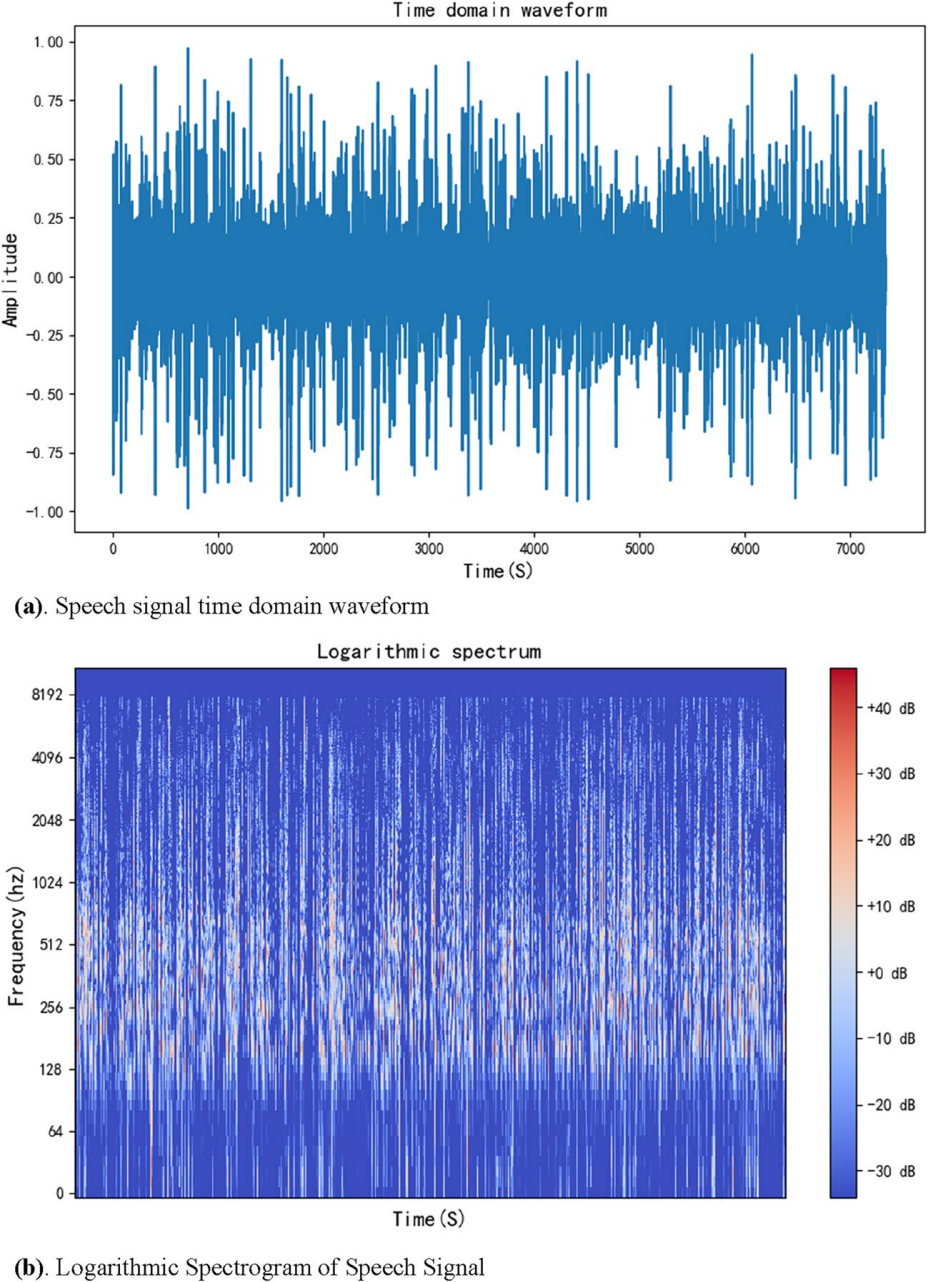
(**a**) The time domain waveform of the audio of the online discussion meeting, the figure contains information about the sound fluctuations of 26 attendees during a 122-min meeting. (**b**) Logarithmic spectrum of the audio from the online discussion session, which contains frequency and sound decibel information for 26 participants over the course of the 122-min session.

Firstly, the video file of the meeting was converted to an audio file in Windows Media Audio format. Speech signal time domain waveform and logarithmic Spectrogram of Speech Signal could be plotted by reading the basic information of the audio file, as shown in Fig. 10.

Secondly, use the Pyhub library in Python to segment the audio file of an online discussion meeting into multiple segments containing only one speaker’s information, as follows:

1. Read the audio file through the AudioSegment function in the PyHub library.
2. Through the PyHub library the silence function can be set by setting the silence audio segmentation threshold, and setting the silence decibel for − 70dBFS, and the silence time for 700 ms.
3. When the sound in the audio is less than − 70dBFS and lasts for more than 700 ms, it will be split from the beginning of the silence, and the split will end at the end of the silence.
4. In the process of audio segmentation, audio segments less than 500 ms long will be discarded.
5. The segmented audio will be saved to a folder to wait for voiceprint recognition.

Finally, use a for loop to iterate through the audio segments in the audio folder that need to be recognized. Use voice recognition to identify the speaker of each audio segment until all audio identities in the folder are recognized. Use the statistics function to get the number of times each speaker has spoken and the total speaking time.

The results of processing and recognizing the audio of the discussion session are shown in Table 5.

**Table 5:** The results of processing and recognizing the audio of the discussion session.

Session length	7340.0 s
Channels	2
Sampling width	2
Sampling frequency	16,000
Sampling points	117,439,542
Total number of segments	2321
Number of recording assistant segments	1680
Identifying the number of segments	1608
Effective segment recognition rate	95.71%
Recording assistant records the total time	6007 s
Total time to identify segments	5752.7 s
Effective time recognition rate	95.76%

The experiment splits the audio file into 2321 segments, and filters and deletes the segments whose lengths are less than 0.7 s, obtaining 1608 audio segments that complete the speaker identification, with a total duration of 5752.7 s. The two recording assistants, by looking at the video replay of the online discussion meeting, came up with a valid audio segment of 1680 and a valid audio duration of 6007 s. In the results of the audio segmentation process, the difference between the number of audios that could identify the speaker and the number provided by the recording assistant was 72, and the difference in duration was 342.74 s. The Recording Assistant performed multiple localizations and playbacks of the 72 audio clips that were not successfully recognized and found that these audio clips had overlapping speech or were interfered with by significant ambient noise, which resulted in unsuccessful speaker recognition.

Overall, the number and duration of recognitions obtained in the experiment matched the results recorded by the recording assistant. The speech parameters obtained by the recording assistant and the speech parameters obtained by the experiment are shown in Table 6:

**Table 6:** Participant activity data.

ID	Assistant records		Experimental results	
	Total duration(s)	Total frequency	Total duration(s)	Total frequency
Host	1060	340	1029.38	326
P1	254	63	246.71	59
P2	141	44	131.2	42
P3	468	148	437.58	139
P4	235	62	220.72	59
P5	58	17	53.59	16
P6	74	22	66.69	20
P7	198	68	180.1	64
P8	32	15	28.29	14
P9	451	109	433.52	104
P10	63	25	59.64	25
P11	169	22	165.85	22
P12	152	24	147.07	23
P13	424	100	408.37	97
P14	121	28	111.5	26
P15	12	6	10.33	6
P16	151	61	135.57	58
P17	101	41	96.23	39
P18	73	31	66.41	27
P19	66	27	61.41	26
P20	122	34	116.53	33
P21	161	57	154.92	57
P22	756	200	744.5	198
P23	440	64	434.87	62
P24	100	15	93.87	13
P25	125	57	117.85	53
sum	6007	1680	5752.7	1608

### Ethics statement

All of our studies involving human participants complied with the Declaration of Helsinki and the ethical standards set by relevant organizations. Our experiments were approved by Xi’an University of Posts and Telecommunications (XUPT) and the Shaanxi Provincial Department of Science and Technology (SPDST), and the use of participants’ audio data for the study was approved by the Ethics Committee of XUPT. All participants were informed of the content of the experiments, and we promise not to disclose the privacy of any participant.

### Analysis of experimental results

Through the two basic indicators of activity, namely, speaking time and speaking frequency, the quantitative score of activity of each participant can be calculated by the activity calculation model. This is because the Host, as the moderator of the meeting, rightfully speaks far more often and for longer than the attendees. If Host’s activity is included in the calculation, it will greatly affect the fairness of other participants’ scores, because, in a real discussion meeting, the activity level of the meeting host must be overactive. So to be able to reflect the activity level of the attendees more objectively, in calculating the activity level, data on the longest speaking time and the longest speaking frequency will be obtained from the 25 participants. The results of the online discussion meeting activity evaluation are shown in Table 7.

**Table 7:** Activity-level statistics (time units: seconds, P: participants).

ID	Total duration(s)	Total frequency	The average duration of each audio segment	Time score	Frequency score	Validity coefficient	Activity Score
Host	1029.38	326	3.16	1.39	1.67	0.88	1.35
P1	246.71	59	4.18	0.32	0.28	1.17	0.35
P2	131.2	42	3.12	0.16	0.19	0.87	0.15
P3	437.58	139	3.15	0.58	0.69	0.88	0.56
P4	220.72	59	3.74	0.29	0.28	1.04	0.29
P5	53.59	16	3.35	0.06	0.05	0.94	0.05
P6	66.69	20	3.33	0.08	0.07	0.93	0.07
P7	180.1	64	2.81	0.23	0.30	0.79	0.21
P8	28.29	14	2.02	0.02	0.04	0.56	0.02
P9	433.52	104	4.17	0.58	0.51	1.16	0.63
P10	59.64	25	2.39	0.07	0.10	0.67	0.06
P11	165.85	22	7.54	0.21	0.08	2.11	0.31
P12	147.07	23	6.39	0.19	0.09	1.79	0.25
P13	408.37	97	4.21	0.54	0.47	1.18	0.60
P14	111.5	26	4.29	0.14	0.10	1.20	0.14
P15	10.33	6	1.72	0.00	0.00	0.48	0.00
P16	135.57	58	2.34	0.17	0.27	0.65	0.14
P17	96.23	39	2.47	0.12	0.17	0.69	0.10
P18	66.41	27	2.46	0.08	0.11	0.69	0.06
P19	61.41	26	2.36	0.07	0.10	0.66	0.06
P20	116.53	33	3.53	0.14	0.14	0.99	0.14
P21	154.92	57	2.72	0.20	0.27	0.76	0.18
P22	744.5	198	3.76	1.00	1.00	1.05	1.05
P23	434.87	62	7.01	0.58	0.29	1.96	0.85
P24	93.87	13	7.22	0.11	0.04	2.02	0.15
P25	117.85	53	2.22	0.15	0.24	0.62	0.12

**Figure 11:**
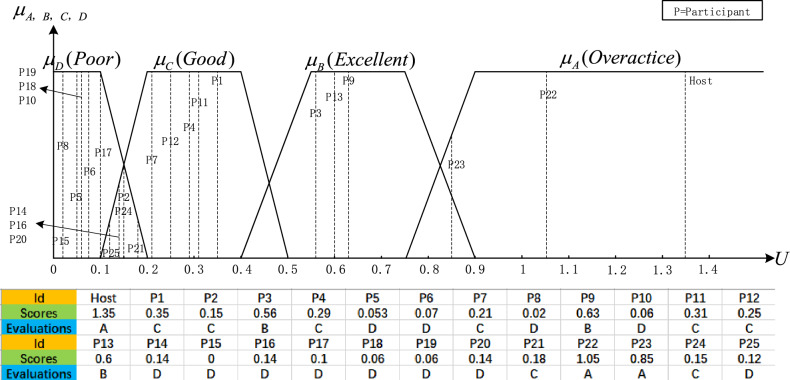
The results of the activity evaluation are: Those rated as “Overactive” were session facilitator Host, participant22, and participant23; Participant3, participant9, and participant13 were rated as “Excellent”, and they spoke much longer and more frequently than the other participants in the discussion session; Participant1, participant2, participant4, participant7, participant11, participant12, participant21 were rated as “Good”, participant24, who spoke for a moderate length and frequency during the discussion session; In contrast, the rest of the participants were rated as “Poor” in terms of activity during the discussion sessions, as they did not speak as often or for as long as they would have liked.

In this experiment, each participant’s activity evaluation results were obtained from four activity membership functions, and the set of activity membership functions is shown in Fig. 11. The results of the activity evaluation are: Those rated as “Overactive” were session facilitator Host, participant22, and participant23; Participant3, participant9, and participant13 were rated as “Excellent”, and they spoke much longer and more frequently than the other participants in the discussion session; Participant1, participant2, participant4, participant7, participant11, participant12, participant21 were rated as “Good”, participant24, who spoke for a moderate length and frequency during the discussion session; In contrast, the rest of the participants were rated as “Poor” in terms of activity during the discussion sessions, as they did not speak as often or for as long as they would have liked.

### Informed consent statement for human subjects research

The research team conducting the study “ ECAPA-TDNN Based Online Discussion Activity -Level Evaluation” hereby confirms that for any experiments involving human participants (including the use of tissue samples), informed consent was obtained from all subjects and/or their legal guardian(s). The consent process was conducted in accordance with the ethical standards set forth in the Declaration of Helsinki, and approval for this research was granted by the Institutional Review Board (IRB) at the following institution: Xi’an University of Posts and Telecommunications, Ethics Committee of Xi’an University of Posts and Telecommunications.

## Final considerations

This project designs a system that applies voiceprint recognition technology and an activity calculation model to online discussion meetings, and then accurately obtains the participants’ activity level in the meeting. The experimental results of the simulated meeting can prove that the problems studied in this paper can be effectively solved by this system, which can effectively capture the identity of the speakers in the meeting and the frequency of their speeches, to obtain feedback on the effect of the online discussion and the evaluation of the participants’ activity-level in the meeting.

This paper has the following areas for improvement in the steps taken to implement the functionality of the system: the recognition accuracy of the voiceprint recognition model can be further improved by improving the algorithm, adding suitable training data, and adjusting the method of data enhancement. The participant’s conference activity-level score should require consideration of the presentation’s relevance to the conference theme.

First, there is still much room for improvement and expansion in the research method and application direction of voiceprint recognition technology. With the research and development of critical technologies of voice recognition, on the one hand, speaker recognition can be applied in classroom discussion, conference communication, and other scenarios that require speaker identification while ensuring stability with improved model accuracy; on the other hand, the training set of audio data can be expanded, and foreign languages and local dialects can be added to make the recognition range more extensive. Second, the activity-level evaluation of online discussion meetings can also be combined with technologies such as facial recognition, attention mechanism, and eye tracking so that the activity-level of participants can be evaluated more objectively and accurately. Secondly, the activity-level assessment of online discussion sessions can also incorporate technologies such as speech-text recognition, face recognition, attention mechanisms, and eye tracking to more objectively and accurately assess the activity-level of participants.

### Supplementary Information

Supplementary Information.

## Data Availability

All data generated or analysed during this study are included in this published article (and its supplementary information files).
